# Plasmonic Geometry-Induced Viscoelastic Biocomplex Formation with Optical Concealment, Liquid Slips, and Soundscapes in Bioassays

**DOI:** 10.1021/acs.analchem.4c04859

**Published:** 2025-03-25

**Authors:** Zoe Bradley, Nikhil Bhalla

**Affiliations:** Nanotechnology and Integrated Bioengineering Centre (NIBEC), School of Engineering, Ulster University, 2-24 York Street, Belfast BT15 1AP, United Kingdom

## Abstract

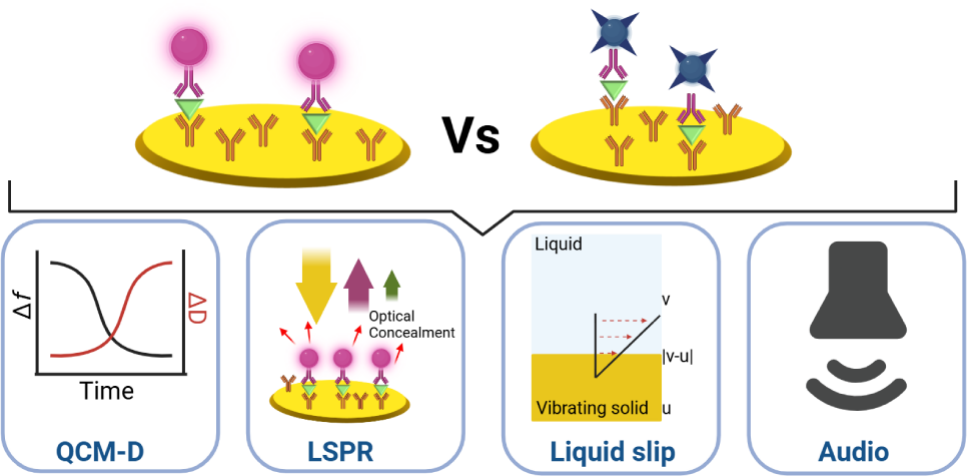

Plasmonic nanoparticles (NPs), typically made up of gold or silver, are widely used in point-of-care bio- and chemical sensing due to their role in enhancing detection sensitivity. Key NP properties influencing sensing performance include the material type, NP size, and geometry. While much research has focused on material and size optimization, less attention has been given to understand NP geometry effects and interactions with biomolecules involved in the bioassay. In this context, we investigate the interfacial properties of the biocomplex formed by spherical-shaped gold nanoparticles (AuNPs) and gold nanostars (AuNSts) during a sandwich assay using localized surface plasmon resonance (LSPR) and quartz crystal microbalance with dissipation (QCM-D). The chosen model to study the biocomplex specifically detects interleukin-6 (IL-6). Our results show that AuNSts, with their anisotropic shape and higher surface area, form antibody–antigen complexes more effectively than AuNPs. AuNSts also create a softer, more hydrated layer due to their complex geometry, which leads to larger liquid slips. Lastly, we showed that AuNSts avoid optical concealment at high IL-6 concentrations, unlike AuNPs, making them more reliable for detecting a wider range of concentrations. These findings highlight the importance of optimizing NP geometry for improved bio/chemical sensor performance.

Noble metal nanoparticles (NPs), primarily made of gold and silver, are a central focus in modern nanotechnology due to their exceptional biocompatibility, low toxicity, and strong affinity for a wide range of biomolecules.^1^ These properties make noble metal NPs particularly valuable for biomedical applications, including drug delivery, imaging, and diagnostics.^2^ The intentional synthesis of spherical NPs dates back to Faraday’s work in the 1850s.^3^ However, it is now well understood that nanomaterials with nonspherical shapes can exhibit unique physical and chemical properties compared to their spherical counterparts.^4^ By simply altering their shape and size, significant enhancements in these properties can be achieved, as different crystal surfaces have varying atom densities and electronic structures.^5^

In the context of point-of-care bio- and chemical sensing, NPs have gained prominence for their ability to enhance detection sensitivity. Essentially, during bioassay optimization, research has traditionally focused on NP size, with studies showing that smaller NPs offer higher sensitivity.^6^ As a result, significant research has been devoted to optimizing NP sizes, but the effect of NP geometry on sensing performance remains underexplored. This gap is critical because NP geometry influences the interaction between NPs and biomolecules in bioassays, potentially impacting the sensitivity and accuracy of detection. Furthermore, NP geometry can improve sensor performance by enhancing the signal-to-noise ratio.

The geometry of NPs is determined by specific synthesis conditions, such as pH, temperature, incubation time, and metal salt concentration, resulting in a variety of shapes, including cubes, rods, triangles, and stars.^7−10^ Gold NPs exhibit a range of colors based on their geometry, size, surrounding dielectric environment, and composition, which influence their plasmonic properties through localized surface plasmon resonance (LSPR). This phenomenon occurs because the plasmonic properties of gold NPs allow for the absorption and scattering of light in the visible spectrum, resulting from the collective oscillation of electrons on the NP surface resonating at specific wavelengths.^11^ For spherical gold NPs (AuNPs), a typical LSPR peak wavelength is seen between 520 and 570 nm for sizes of 20–100 nm in diameter, giving rise to a red color.^12^ While, anisotropic shapes like gold nanostars (AuNSts) have irregular geometry due to sharp tips and protrusions, which give rise to a more complex LSPR, leading to multiple resonance modes. These resonances usually occur at longer wavelengths around 660 nm, producing a blue color. The tips of the AuNSts create “hot spots” where the electromagnetic field is significantly enhanced, shifting the absorption and scattering to different wavelengths compared to AuNPs.^13^ Additionally, the ability of NPs to aggregate, resulting in a color change in the presence of an analyte, is an attractive feature for many researchers, particularly in drug delivery for cancer medicine.^14^

Our work aims to bridge the gap in plasmonic NP geometry research for bioassays by examining how the shape of gold NPs, specifically AuNPs and AuNSts, influences the interfacial properties of biomolecular complexes formed during a sandwich assay. Using LSPR and quartz crystal microbalance with dissipation (QCM-D), our study focuses on detecting interleukin-6 (IL-6) as a model biomolecule. IL-6 is a crucial biomarker in the diagnosis and monitoring of various inflammatory and autoimmune conditions, as well as certain cancers and COVID-19.^15^ Prompt detection is critical for IL-6-related conditions, as early diagnosis and targeted treatment within the first 6 h in response to changing stimuli can greatly improve patient outcomes. Despite its importance, detecting IL-6 at clinically significant concentrations, typically in the pg/mL range, remains challenging and requires a highly optimized bioassay to achieve reliable and sensitive results.^16^ Therefore, NP-based sensors can play a valuable role in monitoring patients with elevated levels of IL-6. Previous studies have used functionalized AuNPs to monitor IL-6 in the pg/mL range with a limit of detection (LOD) of 0.1 pg/mL.^17^ Herein, we use an IL-6 assay as a proof-of-principle system to study the LSPR and QCM-D measurements of AuNPs and AuNSts, investigating how variations in NP geometry influence biosensor performance. By comparison of AuNPs and AuNSts, our work demonstrates that the anisotropic shape and higher surface area of AuNSts lead to more efficient complex formation, a softer and more hydrated interfacial layer, and a broader range of detection without optical concealment. In terms of a sandwich assay, optical concealment is interference that reduces the visibility of the assay’s optical signal, affecting detection accuracy. Using our previously published models on the solid–liquid interface to help understand the properties of the adsorbed layer, we also find liquid slip^18^ associated with biocomplexes. Liquid slip describes the relative velocity differences at the solid–liquid interface, reflecting interfacial dynamics and is integral to understanding sensor performance as it affects resonance parameters, including frequency and dissipation shifts.^19^ Additionally, power spectrum analysis of frequency shifts from QCM-D data is converted into soundscapes. These soundscapes capture the essence of biomolecular interactions, which can also be represented as music or audio signatures, allowing distinct biomolecular interactions to be identified through QCM-D. A schematic of our investigation model, illustrating the measurement techniques, biocomplexes, and key attributes of the study, is presented in Figure 1. Our findings highlight the importance of NP geometry in enhancing the performance of bio/chemical sensors, paving the way for more reliable and sensitive detection methods.

**Figure 1:**
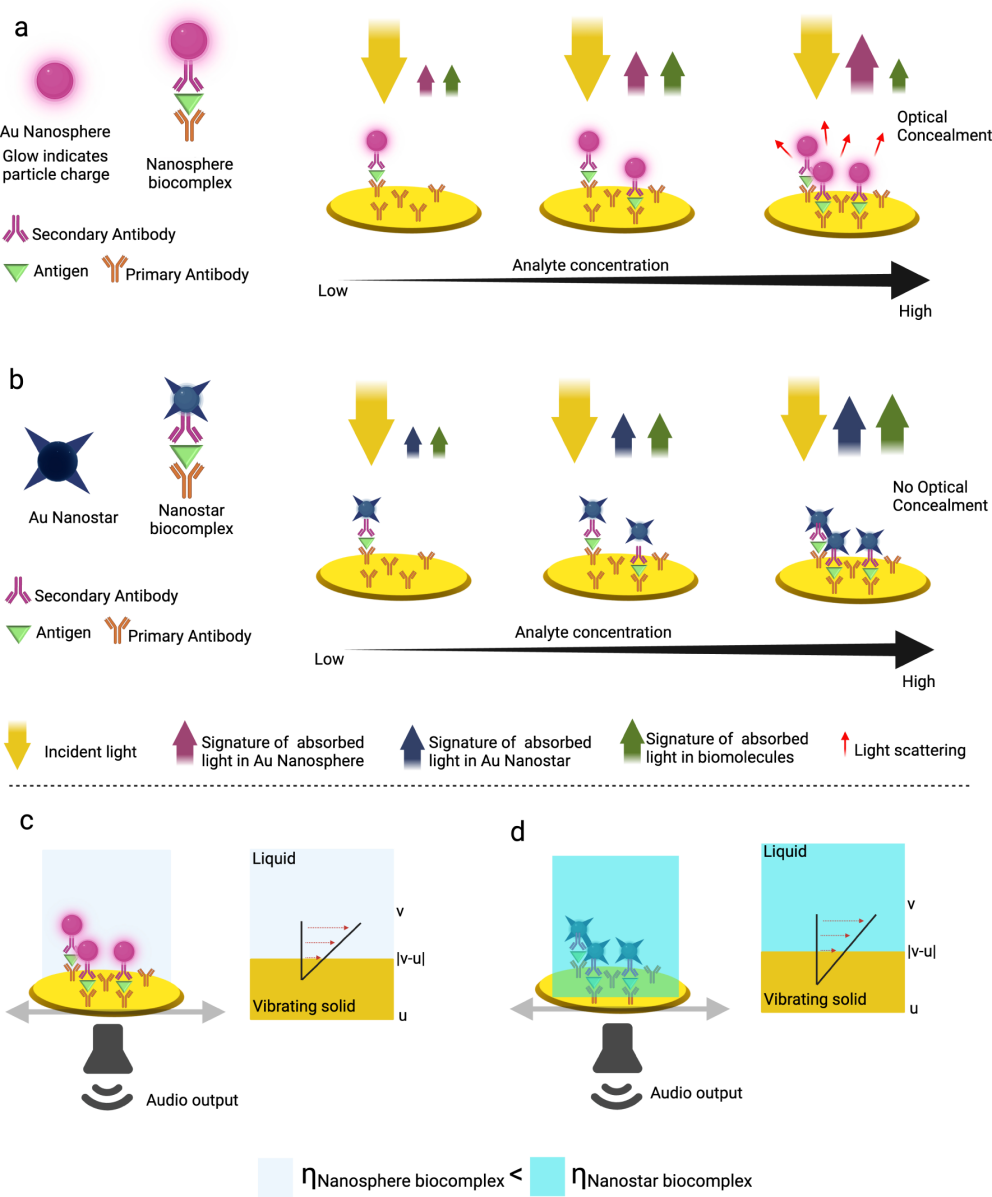
Schematic representation of the study: (a) AuNP biocomplexes formed at low and high analyte concentrations. At high analyte concentrations, the biocomplex exhibits optical concealment. (b) AuNSt biocomplexes formed at low and high analyte concentrations. Unlike AuNPs, the biocomplex at high analyte concentrations shows no optical concealment. (c) A less viscous layer on the QCM-D surface is observed for the AuNP biocomplex, resulting in relatively lower liquid slip. (d) A more viscous layer is formed on the QCM-D surface in the AuNSt biocomplex, leading to relatively higher liquid slip.

## Experimental Section

### Materials

Thiol PEG acetic acid (HS-PEG-COOH, 5 kDa) was purchased from JenKem Technology Ltd. (Beijing, China). Gold (III) chloride hydrate (HAuCl_4_), sodium citrate tribasic dihydrate (Na_3_C_6_H_5_O_7_·2H_2_O, NaCit), hydrochloric acid (HCl), silver nitrate (AgNO_3_), ascorbic acid (C_6_H_8_O_6_, L-AA), 2-(*N*-morpholino)ethanesulfonic acid (C_6_H_13_NO_4_S, MES), *N*-(3-(dimethylamino)propyl)-*N*’-ethylcarbodiimide hydrochloride (C_8_H_17_N_3_·HCl, EDC), *N*-hydroxysuccinimide (C_4_H_5_NO_3_, NHS), 11-mercaptoundecanoic acid (HS(CH_2_)_10_CO_2_H, MUA), 6-mercapto-1-hexanol (HS(CH_2_)_6_OH, MCH), hydroxylamine, Tris-buffered saline (TBS), Tween 20, casein, isopropanol, acetone, and phosphate-buffered saline (PBS) were purchased from Merck Life Science (Dorset, UK). Monoclonal anti-human IL-6 detection antibody (L395), monoclonal anti-human IL-6 capture antibody (L143), and recombinant human IL-6 antigen were purchased from HyTest Ltd. (Turku, Finland). 18.2 MΩ deionized water (dH_2_O) from a Milli-Q Purification System (Sigma-Aldrich, USA) was used throughout the study. All chemicals were used without further modification unless specified.

### Gold Nanoparticle Synthesis

AuNPs of 20 nm diameter were synthesized using the conventional Turkevich method.^20^ First, 50 mL of 1 mM HAuCl_4_ was heated to 100 °C under continuous stirring. Then, 3 mL of 38 mM NaCit was added, and a color change from yellow to black to dark red was observed. The 20 nm AuNPs solution was allowed to cool to room temperature under continuous stirring. Finally, AuNPs were washed twice by centrifuging at 3600 *g* for 10 min, the supernatant was removed, and the pellet was resuspended with dH_2_O.

### Gold Nanostar Synthesis

AuNSts were synthesized using a protocol reported by Lou-Franco et al., with minor modifications.^21^ Briefly, 25 μL of 100 mM HAuCl_4_ and 5 μL of 2 M HCl were added to 10 mL of dH_2_O under continuous stirring. Then, 75 μL of 20 nm AuNPs, 100 μL of 3 mM AgNO_3_, and 50 μL of 100 mM L-AA were added simultaneously to the solution under continuous stirring. A color change from pale yellow to blue was observed, and 110 μL of 1 mM NaCit was added to stabilize the particles. The AuNSts were then washed twice by centrifuging at 4180 *g* for 35 min, the supernatant was removed, and the pellet was resuspended with dH_2_O.

### Nanoparticle Functionalization

NPs were functionalized with a carboxyl layer by incubating them with 5 mM HS-PEG-COOH, away from light and under continuous rotation for 2 h. The carboxylated NPs were then washed twice by centrifugation at 10600 *g* for 30 min. After each centrifugation, the supernatant was removed, and the pellet was resuspended in dH_2_O.

### Covalent Conjugation

Functionalized NPs were conjugated by mixing 500 μL of carboxylated NPs with 200 μL of 0.1 mg/mL detection antibody in 10 mM MES, 100 μL of 100 mM MES, 200 μL of 1 mM EDC, and 400 μL of 1 mM NHS. The solution was incubated under continuous stirring for 20 min. Following this, 10 μL of hydroxylamine was added to quench the solution and incubated for a further 10 min. Then, 10 mL of TBS + 0.05% Tween 20 was added, and the solution was centrifuged for 20 min at 10600 *g*. The supernatant was removed, and the final conjugate was resuspended with TBS + 0.5% Tween 20 + 1% casein.

### Characterization

NP charge was determined by dynamic light scattering (DLS) using a NanoZetasizer ZS Series (Malvern Panalytical, UK). UV–vis absorption spectra of NPs and their conjugates were obtained using a LAMBDA 365 spectrometer (PerkinElmer, UK). The spectra were recorded from 1100 to 190 nm at a 120 nm/min scan speed in disposable cuvettes with a 1 cm optical path length at room temperature. The NP size, structure, monodispersity, and the quartz crystal surface were studied using a Hitachi SU5000 scanning electron microscope (SEM) equipped with an Oxford Instruments Energy-Dispersive X-ray Analysis (EDX) system. Following this, ImageJ software was used to determine the NP diameter.

### Quartz-Crystal Microbalance with Dissipation (QCM-D) Immobilization and Monitoring

QCM-D measurements were performed with an OpenQCM Next system (Open QCM, Pompeii, Italy) that monitors the changes in the resonance frequency Δ*f* and the energy dissipation factor Δ*D*. AT-cut quartz crystals with a Au coating (a diameter of 14 mm, a thickness of 0.16 mm, and a resonance frequency of 10 MHz) were used. The quartz crystal casing comprised an aluminum anodized shell with an SiO_2_ optical window, making it possible to integrate LSPR; see Figure S3. The QCM-D crystals were first cleaned by sonicating with acetone for 5 min, followed by rinsing with isopropanol and dH_2_O.

For capturing antibody immobilization onto the quartz crystal surface, a 100 μL solution containing 1 mM MUA and MCH in a ratio of 1:9, respectively, was pipetted onto a quartz crystal and incubated in a humidity chamber for 18 h. Subsequently, the quartz crystal was rinsed with 10 mM PBS at pH 7.4 and treated with 10 mM EDC/NHS in a ratio of 4:1, respectively, and incubated for 20 min. Following this step, 60 μL of 1 mg/mL capture antibody diluted in 10 mM PBS at pH 7.4 was pipetted onto the quartz crystal and incubated for 15 min. Finally, the reaction was quenched with 1 mM ethanolamine hydrochloride before use.

### Localized Surface Plasmon Resonance

The LSPR response was analyzed using a custom-built optical setup comprising components for sample illumination and light collection. This configuration is similar to those employed in earlier studies.^22,23^ The system incorporated two fiber optic cables, one linked to a light source (DH-mini-UV–vis–NIR) and another to a spectrometer, all sourced from Ocean Optics (now Ocean Insight, The Netherlands). An RTL-T stage, also from Ocean Optics, facilitated the alignment of fiber optics, and, before signal acquisition, the spectrometer underwent dark and light spectrum calibration. OceanView software was used to observe and record the wavelength-dependent light absorption by nanostructures in absorption mode, enabling LSPR signal detection.

## Results and Discussion

Initial surface characterization of NPs and the quartz crystal surface was completed using SEM and UV–vis spectroscopy. Figure S1a,b shows the UV–vis spectrum for citrate-capped (referred to as bare herein) NPs, NPs functionalized with a carboxyl layer, and NPs conjugated with a detection antibody. For both types of NPs, successful surface functionalization with the carboxyl layer is indicated by a red shift in wavelength.^24^ For AuNPs, a mean red shift of 2 nm is observed following carboxyl functionalization, whereas for AuNSts, the mean red shift is 88 nm after the same modification. Following this, the successful conjugation of both NPs is indicated by a protein peak at 280 nm. Figure S1c,d shows the SEM images of AuNPs and AuNSts with mean diameters of 30 and 70 nm, respectively. Figure S2a shows the scanning electron microscopy (SEM) characterization of the quartz crystal surface with gold nanostructures of 60 nm average diameter, and Figure S2b presents EDX spectra displaying the elemental component analysis, indicating a primary gold composition.

**Figure 2:**
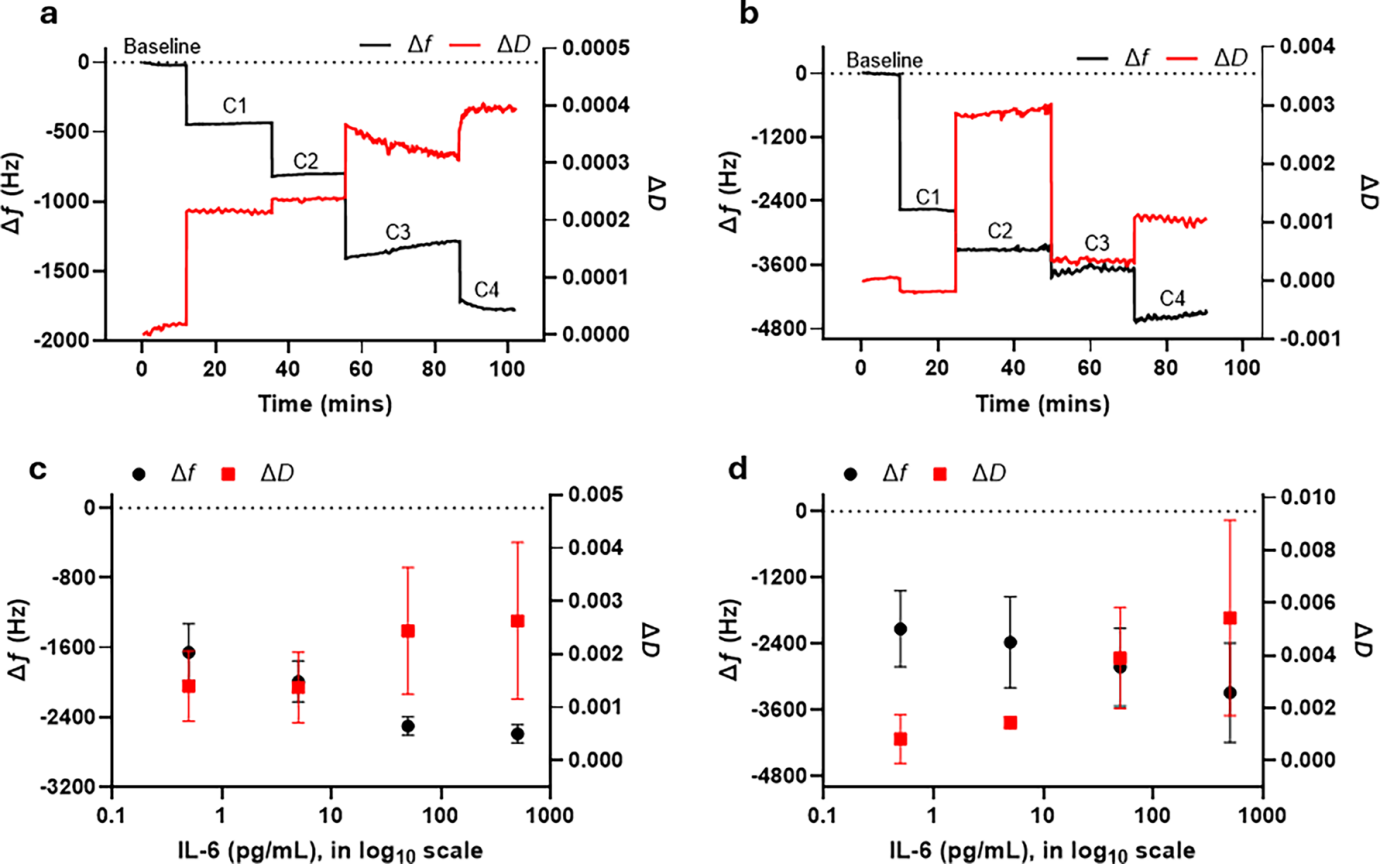
QCM**-**D frequency and dissipation shifts. (a) Real-time AuNPs binding, (b) real-time AuNSts binding, (c) AuNPs average shifts on the QCM-D surface, and (d) AuNSts average shifts on the QCM-D surface. Error bars in c and d display standard deviations *n* ≥ 3, where *n* is the number of samples. C1, C2, C3, and C4 refer to the concentrations of the IL-6 antigen, which are 0.5, 5, 50, and 500 pg/mL, respectively.

To measure changes in frequency and dissipation, we used a QCM-D and quartz crystal as the piezoelectric material, which was immobilized with a capture antibody and IL-6 antigen. The conjugate, consisting of a detection antibody labeled with NPs in 10 mM PBS, was used as the liquid for both real-time and static experiments. Real-time measurements were used to observe the binding kinetics of the antigen–antibody complex as they occurred. For static measurements, results were collected after a 1 h incubation of the antibody–antigen complex at each concentration to ensure proper QCM-D stabilization and sufficient reaction time. C1, C2, C3, and C4 refer to the concentrations of the IL-6 antigen, which are 0.5, 5, 50, and 500 pg/mL, respectively, and the baseline used was 10 mM PBS, pH 7.4. Figure 2a,b displays the real-time measurements for AuNPs and AuNSts, while Figure 2c,d shows the static measurements for AuNPs and AuNSts. A greater change in frequency is observed in both real-time and static measurements for AuNSts compared to AuNPs. This is attributed to the higher surface area-to-volume ratio of the AuNSts. The sharp tips and surface roughness of AuNSts facilitate the formation of the antibody–antigen complex, resulting in greater mass per unit and a larger frequency change. However, a more uniform change in dissipation is displayed by AuNPs, as the spherical shape allows for an even charge distribution across the particle surface, leading to consistent electrostatic interactions with the sensor surface and the bioconjugate, which refers to functionalized NPs conjugated with detection antibodies.^25^ This theory is supported by our charge measurements in Table S1, where the AuNPs bioconjugate has a zeta potential of −11.7 ± 0.558 mV, and the AuNSts bioconjugate has a zeta potential of −10.3 ± 0.207 mV. See the Supporting Information for the results of a *t*-test which achieved a statistically significant *p*-value of 0.0002, suggesting that a higher zeta potential value (higher charge) indicates greater repulsion between particles, leading to increased stability.

Linear regression analysis in Figure S4 demonstrates significantly greater sensitivity for AuNSts compared to AuNPs in detecting changes in frequency. Sensitivity, calculated as the slope of the concentration-dependent curve, was remarkably higher for AuNSts (−393.2 Hz per unit concentration, pg/mL) compared to AuNPs (−331.8 Hz/pg/mL). These results underscore the superior responsiveness of AuNSts to frequency changes. Furthermore, based on the frequency shift of the baseline and the magnitude of sensitivity, the study achieved comparable limits of detection (LODs) for AuNPs and AuNSts: 0.059 and 0.061 pg/mL, respectively (see the Supporting Information for the LOD equation). Although large standard deviations in the measurements for AuNSts contribute to a slightly elevated LOD, this does not undermine their superior performance in terms of frequency shift and sensitivity. While the LOD values are comparable, AuNSts exhibit much greater frequency shifts and significantly higher sensitivity compared to AuNPs.

**Figure 3:**
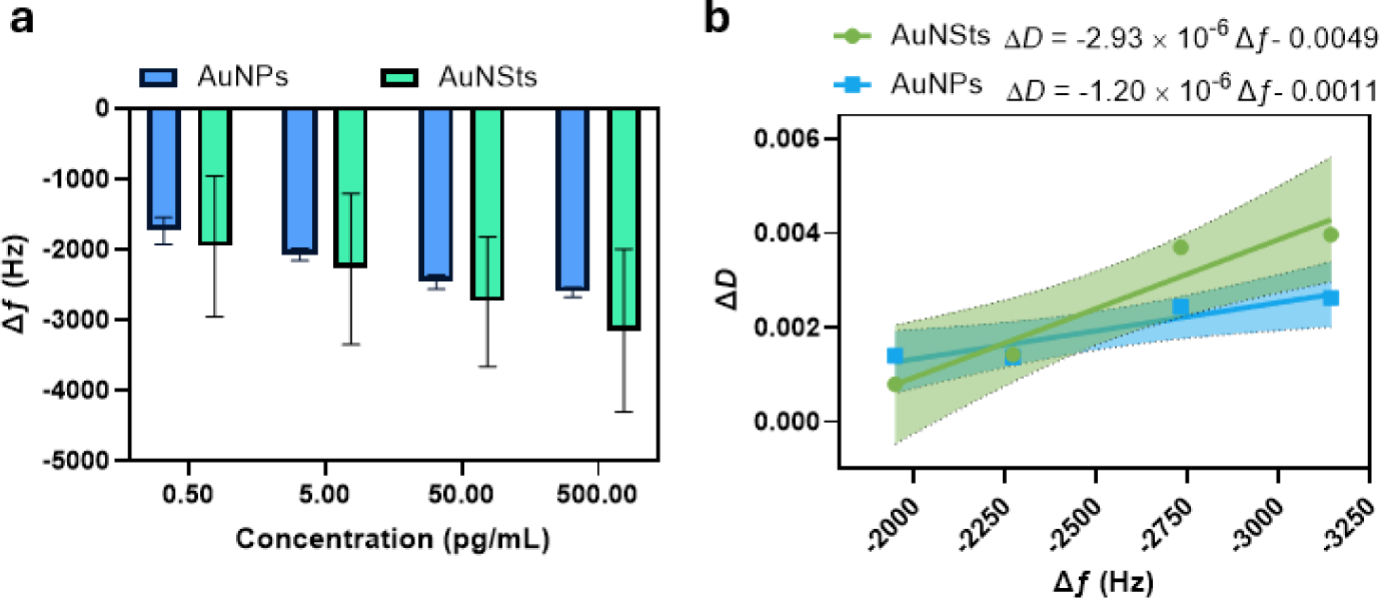
Sensitivity and viscoelasticity of AuNPs and AuNSts. (a) Sensor change in frequency versus concentration of IL-6 displaying sensitivity. A more sensitive result is proportional to a larger frequency shift. (b) Sensor change in dissipation versus frequency. A higher slope indicates a more viscoelastic layer.

We also used changes in frequency and dissipation to further understand the sensitivity and viscoelastic properties of the sensor. As seen in Figure 3a, the frequency shift is proportional to the increasing IL-6 concentration for both AuNPs and AuNSts, with AuNSts displaying a greater frequency shift and, therefore, higher sensor sensitivity. However, we observed that AuNSts exhibit larger error bars compared to those of AuNPs, indicating greater variability in the measurements. This variability is likely due to the non-uniform shape of the AuNSts, which can result in inconsistent particle behavior across different measurements. Despite this variability, AuNSts demonstrate promising performance in terms of detection sensitivity. The larger error bars for AuNSts may influence the detection limit, and in certain cases, these star-shaped particles may be at a disadvantage relative to AuNPs, which show smaller error bars and thus more consistent behavior.

In QCM-D measurements, a larger frequency shift indicates greater sensitivity because it reflects a more significant change in mass on the crystal surface and, therefore, more formations of the antibody–antigen complex. The Sauerbrey equation shows that the frequency increases proportionally with added mass; however, this equation is only applicable to a uniform, evenly covered, rigid, and thin film that vibrates in phase with the crystal surface.^26^ Instead, the Kelvin–Voigt model, which considers viscoelastic materials that exhibit both solid-like and liquid-like behavior, is more suitable for NPs.^27^ A larger shift means that even small mass changes, such as those from molecular interactions, cause noticeable frequency changes. This improves the detection of low concentrations or subtle variations in the sample. Additionally, larger frequency shifts enhance the signal-to-noise ratio, making it easier to distinguish the signal from background noise and thus increasing the sensor’s sensitivity.

The slope of the change in dissipation versus frequency change, as shown in Figure 3b reflects the viscoelastic properties of the absorbed layers at the sensor surface. AuNSts have a higher slope, indicating a more viscoelastic and softer layer compared to AuNPs. AuNSts exhibit more viscoelastic behavior compared to AuNPs due to their complex geometry, which provides increased flexibility and deformability. The irregular shapes of AuNSts also aid the adsorption of more molecules, forming a softer and more hydrated layer on the sensor surface. In contrast, the uniform, spherical shape of AuNPs results in less surface interaction and simpler, more rigid interfacial behavior.

It can also be noted that as the concentration of IL-6 increases, the frequency change and energy dissipation also generally increase due to the presence of fluid inertia. Fluid inertia is closely linked to the attraction between the fluid and the surface, which means that liquid slip on the surface can significantly impact resonance parameters.^28^ Consequently, slip is anticipated at the interface, with the magnitude of slip influenced by the interaction between the liquid and the solid surface in both real-time and static measurements. Essentially, liquid slip affects both the frequency shift and energy dissipation, particularly when the slip length is comparable to the inertial length of the liquid.^18^ The liquid slip with inertia is calculated using eq 1:
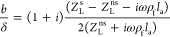
1

Where ρ_f_ is the fluid with density,  is the slip impedance,  is the no-slip impedance, ω is the frequency of the vibrating solid, *b* is the slip length, *l*_*a*_ is the inertia length of the solid–liquid interface (0.3 nm), and δ is the characteristic decay length of the fluid, also known as the penetration length. The dimensionless real and imaginary parts of *b*/δ are used to calculate the magnitude using eq 2:
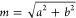
2

**Figure 4:**
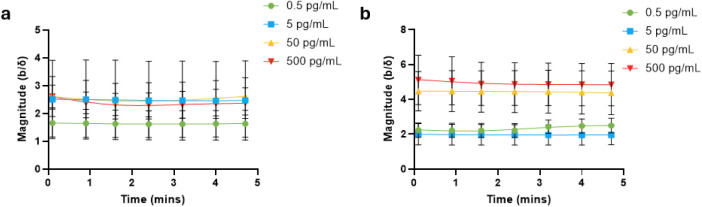
Liquid slip with the inertia model. (a) Magnitude of AuNPs liquid slip. (b) Magnitude of AuNSts liquid slip. a and b are fitted with spline fitting.

Where *m* is the magnitude of liquid slip with inertia, *a* is the real part, and *b* is the imaginary part. Figure 4a,b shows the relationship between the IL-6 concentration and normalized slip with inertia over time in terms of magnitude (the magnitude represents the slip magnitude, which reflects the degree of liquid slip at the solid–liquid interface). Figure 4 shows that overall, AuNSts have a greater magnitude compared to AuNPs, and as IL-6 concentration increases, so does the liquid slip with inertia magnitude. A greater magnitude indicates that the liquid is experiencing less resistance when moving over a surface due to the more viscoelastic properties displayed by AuNSts. From Figure 4a,b, we can also determine that slip values do not change for an individual concentration tested over time for both AuNPs and AuNSts.

**Figure 5:**
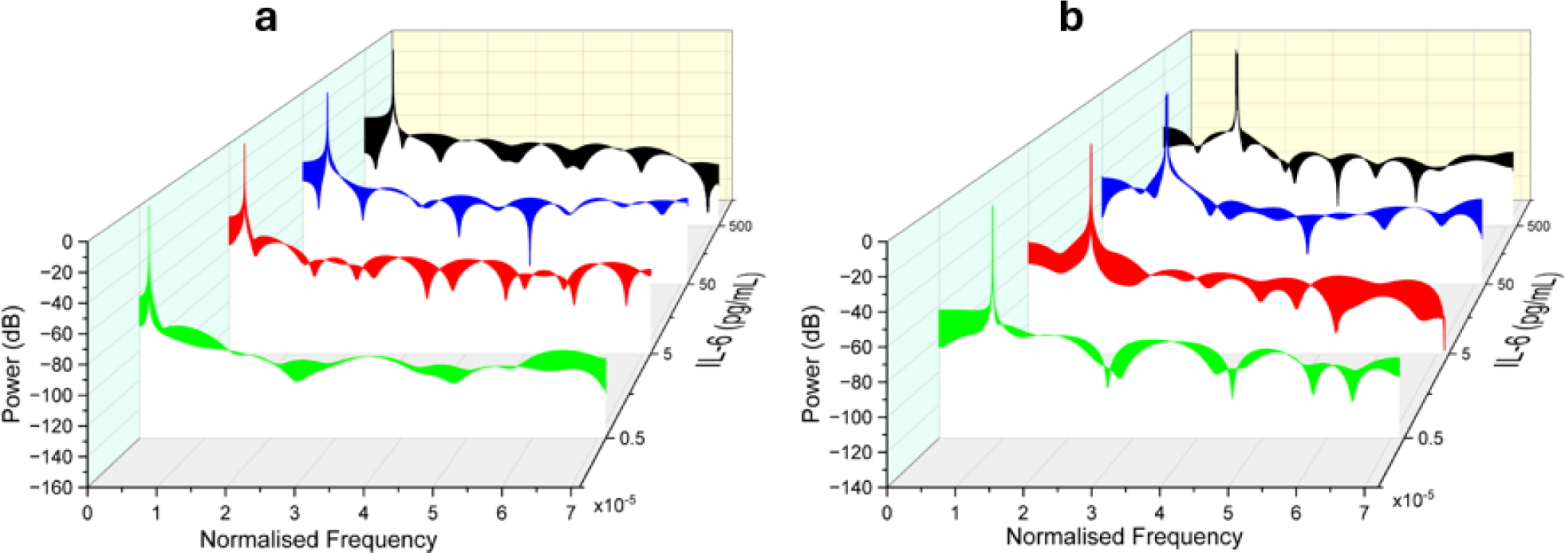
Sensor periodogram soundscapes of (a) AuNPs and (b) AuNSts. The soundscapes were obtained from real-time changes in frequency.

Next, we performed power spectrum analysis on the QCM-D data of real-time measurements, as seen in Figure 5a,b. Power spectrum analysis in the form of periodograms displays unique soundscapes for AuNPs and AuNSts and was calculated from 20 consecutive frequency shift values for each analyte concentration. Normalized frequency (*F*/Fs) was calculated by dividing the frequency (*F*) by the sampling frequency of 44100 Hz (Fs). Tones were generated for each frequency for each second and concatenated using eq 3.
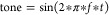
3

Where *f* is the frequency shift and *t* is the time vector for each second. Power was normalized by converting units to decibels (dB) using eq 4.
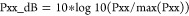
4

Where Pxx_dB is the power with respect to dB, Pxx is the power, and max(Pxx) is the maximum power.

**Figure 6:**
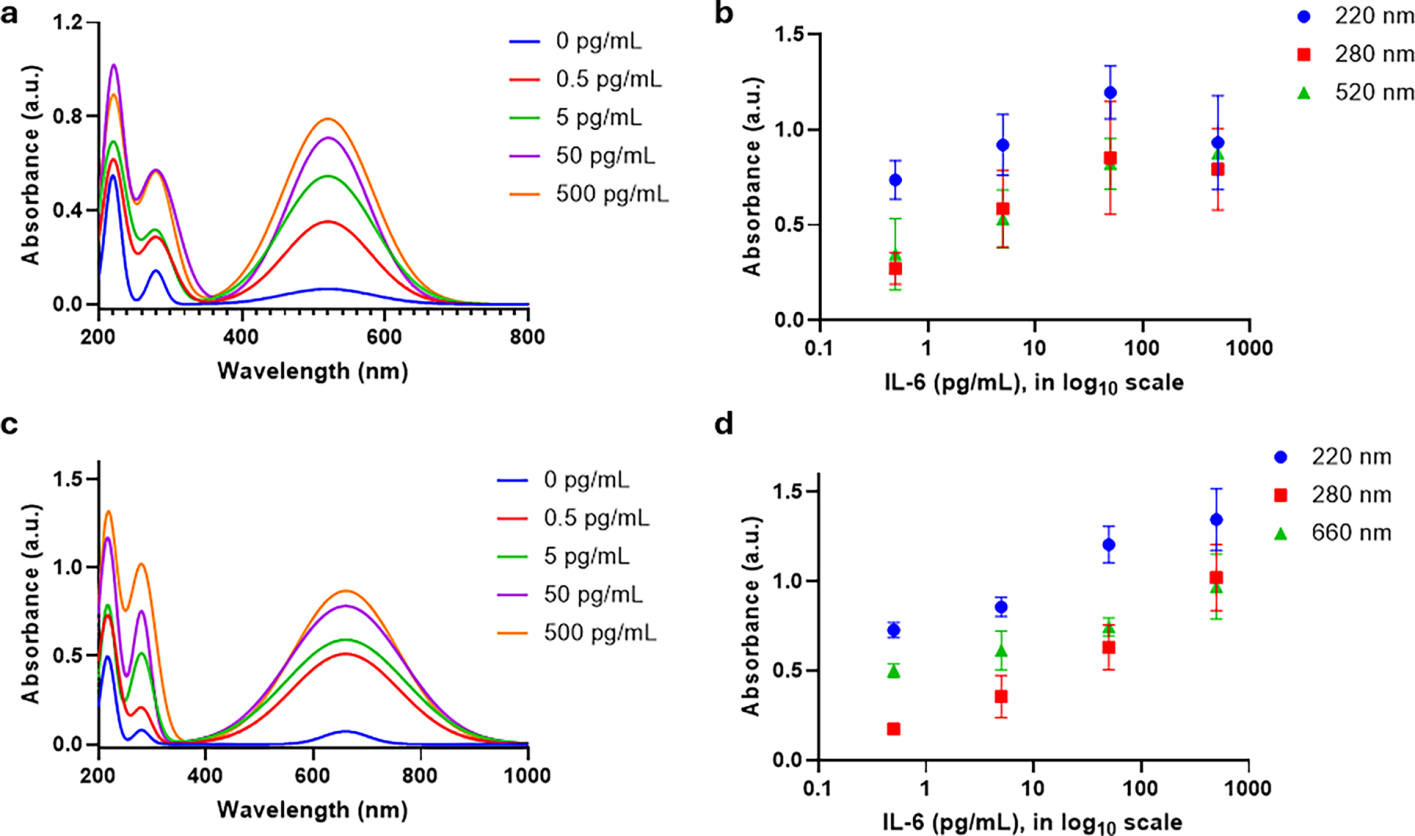
LSPR characterization. (a) AuNPs spectrum, (b) AuNPs peak absorbance versus concentration, (c) AuNSts spectrum, and (d) AuNSts peak absorbance versus concentration. Error bars in (b) and (d) display standard deviations.

Figure 6a,c shows the LSPR absorbance spectra for AuNPs and AuNSts, respectively. In both spectra, the peaks in the 190–280 nm region are attributed to the presence of aromatic amino acids, particularly tryptophan and tyrosine, in IL-6. The peak observed at 520 nm in Figure 6a corresponds to the presence of the AuNPs. As the IL-6 concentration increases from 50 pg/mL to 500 pg/mL, there is a decrease in absorbance due to the saturation of AuNPs, which causes optical concealment. In contrast, Figure 6c shows that AuNSts do not exhibit this trend. Instead, there is a constant increase in absorbance as the IL-6 concentration rises. Figure 6b,d illustrates the peak absorbance versus IL-6 concentration for AuNPs and AuNSts, respectively. The optical concealment trend observed with AuNPs results in a decline in absorbance beyond saturation, limiting the response range to 0–50 pg/mL, whereas AuNSts maintain a response range of 0–500 pg/mL.

**Figure 7:**
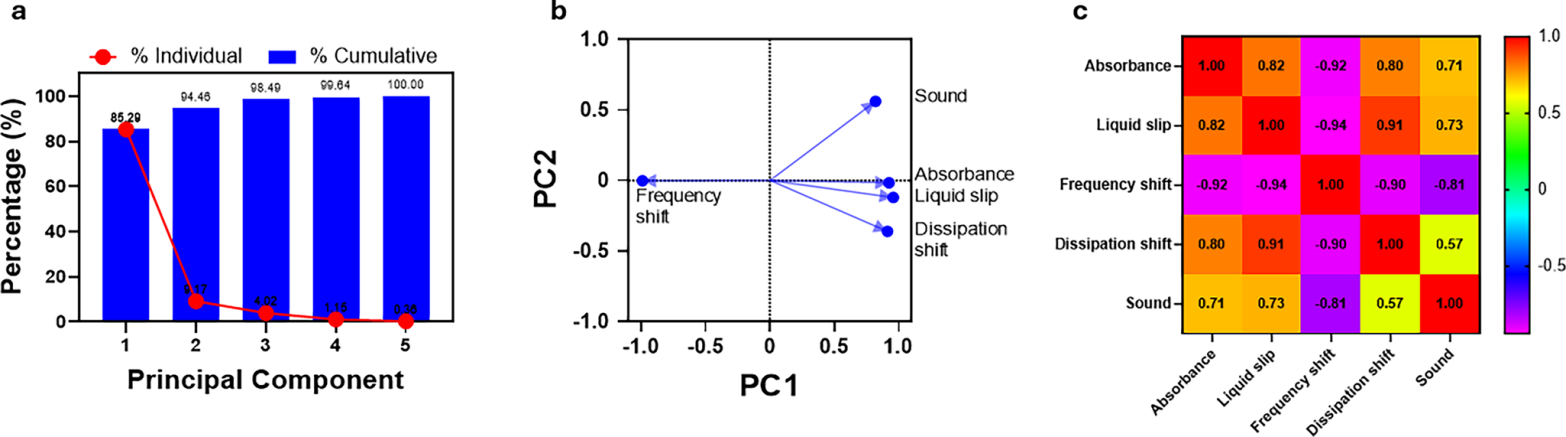
Principal component analysis of analytical techniques. (a) Proportion of variance demonstrates the contribution of each principal component toward the variance in the given data. The principal components 1 and 2 having a collective variance of 94.46%, (b) a loading plot showing PC1 and PC2, and (c) a correlation matrix showing Pearson correlation (*r*) between all variables used within the PCA.

We also analyzed the analytical techniques used by principal component analysis (PCA), see Figure 7, with the aim of showing a clear trend between variables.^29^ We performed PCA using the multiple variable analysis tool built into GraphPad Prism 10 software.^22^ From Figure 7a, it is evident that PC1 and PC2 account for 94.46% of the variance in the data, and these components were used for further analysis. A loading plot (Figure 7b) illustrates that points closer to each other represent closely related parameters. More specifically, if two points lie along the same line, one parameter can be considered redundant in describing the behavior of the analyzed system. Within this context, it can be observed that the frequency shift has an inverse relationship with all other variables, including the dissipation shift, as expected. Additionally, dissipation shift, absorbance, sound, and liquid slip are directly related, a finding further supported by the correlation matrix shown in Figure 7c.

## Conclusion

This study provides critical insights into the influence of NP geometry, highlighting the enhanced performance of AuNSts over AuNPs for IL-6 detection. The distinctive properties of AuNSts, characterized by their complex geometry with sharp tips, result in multiple resonance modes and significant electromagnetic field enhancement, leading to superior biosensor sensitivity. AuNSts exhibit greater frequency shifts in both real-time and static QCM-D measurements compared to AuNPs. This enhanced sensitivity is attributed to the higher surface area-to-volume ratio and increased surface roughness of AuNSts, which better facilitate the formation of the antibody–antigen complex by allowing more analytes to be captured. However, AuNPs display more uniform dissipation due to a greater charge, resulting in a more stable colloid compared to AuNSts. The study also highlighted the viscoelastic properties of the absorbed layers on the sensor surface. AuNSts demonstrated more viscoelastic behavior due to their complex geometry, forming a softer and more hydrated layer on the sensor surface. Moreover, the investigation into liquid slip dynamics revealed that AuNSts showed greater changes due to their greater viscoelastic properties compared to AuNPs.

Furthermore, the LSPR absorbance spectra indicated optical concealment in AuNPs due to NP saturation at higher IL-6 concentrations, a phenomenon not observed in AuNSts. This optical concealment trend in AuNPs suggests a limit to their effectiveness at higher analyte concentrations, whereas AuNSts maintain consistent absorbance, making them more reliable for detecting a broader range of IL-6 concentrations. Overall, this study highlights the critical influence of NP spherical geometry on biosensor performance, demonstrating that AuNSts offer significant advantages over AuNPs in terms of sensitivity, viscoelastic properties, and optical detection. These findings pave the way for developing more effective and reliable biosensors for various medical and diagnostic applications, emphasizing the importance of NP shape in optimizing biosensor functionality.

## Supporting Information Available

Audio files extracted from the periodogram soundscapes for AuNPs (C1, C2, C3, and C4) and AuNSts (C1, C2, C3, and C4) correspond to IL-6 concentrations of 0.5, 5, 50, and 500 pg/mL, respectively (ZIP)
Bioassay characterization; QCM-D surface characterization; QCM-D frequency shift with linear regression analysis; charge measurements; statistical analysis; details on limit of detection calculation (PDF)

## References

[ref1] Singh H.; Bamrah A.; Bhardwaj S. K.; Deep A.; Khatri M.; Brown R. J.; Bhardwaj N.; Kim K.-H. Recent advances in the application of noble metal nanoparticles in colorimetric sensors for lead ions. Environ. Sci.: nano. 2021, 8, 863–889. 10.1039/D0EN00963F.

[ref2] Hou F.; Sun S.; Abdullah S. W.; Tang Y.; Li X.; Guo H. The application of nanoparticles in point-of-care testing (POCT) immunoassays. Anal. Methods. 2023, 15, 2154–2180. 10.1039/D3AY00182B. 37114768

[ref3] Mingos D. M. P. Historical Introduction to Gold Colloids, Clusters and Nanoparticles. In Gold Clusters, Colloids And Nanoparticles I; 2014, pp. 1–47.

[ref4] Pearce A. K.; Wilks T. R.; Arno M. C.; O’Reilly R. K. Synthesis and applications of anisotropic nanoparticles with precisely defined dimensions. Nat. Rev. Chem. 2021, 5, 21–45. 10.1038/s41570-020-00232-7. 37118104

[ref5] Ielo I.; Rando G.; Giacobello F.; Sfameni S.; Castellano A.; Galletta M.; Drommi D.; Rosace G.; Plutino M. R. Synthesis, chemical-physical characterization, and biomedical applications of functional gold nanoparticles: A review. Molecules 2021, 26, 5823 10.3390/molecules26195823. 34641367 PMC8510367

[ref6] Bradley Z.; Cunningham D.; Bhalla N. Refractive index-modulated LSPR sensing in 20–120 nm gold and silver nanoparticles: a simulation study. ECS Sens. Plus 2023, 2, 043402 10.1149/2754-2726/ad08d8.

[ref7] Sun Y.; Zhao Z.; Zhou R.; Li P.; Zhang W.; Suematsu K.; Hu J. Synthesis of In2O3 nanocubes, nanocube clusters, and nanocubes-embedded Au nanoparticles for conductometric CO sensors. Sens. Actuators, B 2021, 345, 130433 10.1016/j.snb.2021.130433.

[ref8] Zhang Q.; Yu Y.; Yun X.; Luo B.; Jiang H.; Chen C.; Wang S.; Min D. Multicolor colorimetric sensor for detection of omethoate based on the inhibition of the enzyme-induced metallization of gold nanorods. ACS Appl. Nano Mater. 2020, 3, 5212–5219. 10.1021/acsanm.0c00641.

[ref9] Koetz J. The Effect of Surface Modification of Gold Nanotriangles for Surface-Enhanced Raman Scattering Performance. Nanomaterials 2020, 10, 2187 10.3390/nano10112187. 33147806 PMC7694140

[ref10] Lu D.; Zhu D. Z.; Gan H.; Yao Z.; Luo J.; Yu S.; Kurup P. An ultra-sensitive molecularly imprinted polymer (MIP) and gold nanostars (AuNS) modified voltammetric sensor for facile detection of perfluorooctance sulfonate (PFOS) in drinking water. Sens. Actuators, B 2022, 352, 131055 10.1016/j.snb.2021.131055.

[ref11] Babicheva V. E. Optical processes behind plasmonic applications. Nanomaterials 2023, 13, 1270 10.3390/nano13071270. 37049363 PMC10097005

[ref12] Pluchery O.; Prado Y.; Watkins W. A complete explanation of the plasmonic colours of gold nanoparticles and of the bichromatic effect. J. Mater. Chem. C 2023, 11, 15824–15832. 10.1039/D3TC02669H.

[ref13] Atta S.; Canning A. J.; Vo-Dinh T. A simple low-cost flexible plasmonic patch based on spiky gold nanostars for ultra-sensitive SERS sensing. Analyst 2024, 149, 2084–2096. 10.1039/D3AN02246C. 38415724 PMC12768891

[ref14] Yu W.; Shevtsov M.; Chen X.; Gao H. Advances in aggregatable nanoparticles for tumor-targeted drug delivery. Chin. Chem. Lett. 2020, 31, 1366–1374. 10.1016/j.cclet.2020.02.036.

[ref15] McCrae L. E.; Ting W.-T.; Howlader M. M. Advancing electrochemical biosensors for interleukin-6 detection. Biosens. Bioelectron.: x 2023, 13, 100288 10.1016/j.biosx.2022.100288.

[ref16] Adrover-Jaume C.; Alba-Patino A.; Clemente A.; Santopolo G.; Vaquer A.; Russell S. M.; Baron E.; Campo M. D. M. G. D.; Ferrer J. M.; Berman-Riu M.; et al. Paper biosensors for detecting elevated IL-6 levels in blood and respiratory samples from COVID-19 patients. Sens. Actuators, B 2021, 330, 129333 10.1016/j.snb.2020.129333. PMC7833127 33519090

[ref17] Alba-Patino A.; Russell S. M.; Borges M.; Pazos-Pérez N.; Álvarez Puebla R. A.; de la Rica R. Nanoparticle-based mobile biosensors for the rapid detection of sepsis biomarkers in whole blood. Nanoscale Adv. 2020, 2, 1253–1260. 10.1039/D0NA00026D. 36133049 PMC9418776

[ref18] Payam A. F.; Kim B.; Lee D.; Bhalla N. Unraveling the liquid gliding on vibrating solid liquid interfaces with dynamic nanoslip enactment. Nat. Commun. 2022, 13 (1), 6608 10.1038/s41467-022-34319-0. 36329039 PMC9633805

[ref19] Chen Q.; Xu S.; Liu Q.; Masliyah J.; Xu Z. QCM-D study of nanoparticle interactions. Adv. Colloid Interface Sci. 2016, 233, 94–114. 10.1016/j.cis.2015.10.004. 26546115

[ref20] Kimling J.; Maier M.; Okenve B.; Kotaidis V.; Ballot H.; Plech A. Turkevich method for gold nanoparticle synthesis revisited. J. Phys. Chem. B 2006, 110, 15700–15707. 10.1021/jp061667w. 16898714

[ref21] Lou-Franco J.; Zhao Y.; Nelis J. L.; Stewart L.; Rafferty K.; Elliott C.; Cao C. Smartphone-based immunochemical sensor exploiting peroxidase-like activity of ligand-capped gold nanostars: a proof-of-concept detection of Mycobacterium bovis. Biosens. Bioelectron. 2023, 220, 114857 10.1016/j.bios.2022.114857. 36335710

[ref22] Bhalla N. Measurement of Human Urine Specific Gravity Using Nanoplasmonics: A Paradigm Shift from Scales to Biosensors. Adv. Sens. Res. 2024, 3 (4), 2300115 10.1002/adsr.202300115.

[ref23] Bhalla N.; Lee D.; Sathish S.; Shen A. Q. Dual-mode refractive index and charge sensing to investigate complex surface chemistry on nanostructures. Nanoscale 2017, 9, 547–554. 10.1039/C6NR07664E. 27892593

[ref24] Retout M.; Blond P.; Jabin I.; Bruylants G. Ultrastable PEGylated calixarene-coated gold nanoparticles with a tunable bioconjugation density for biosensing applications. Bioconjugate Chem. 2021, 32, 290–300. 10.1021/acs.bioconjchem.0c00669. 33439626

[ref25] Zhang L.; Mazouzi Y.; Salmain M.; Liedberg B.; Boujday S. Antibody-gold nanoparticle bioconjugates for biosensors: synthesis, characterization and selected applications. Biosens. Bioelectron. 2020, 165, 112370 10.1016/j.bios.2020.112370. 32729502

[ref26] Kravchenko S.; Snopok B. “Vanishing mass” in the Sauerbrey world: quartz crystal microbalance study of self-assembled monolayers based on a tripod-branched structure with tuneable molecular flexibility. Analyst 2020, 145, 656–666. 10.1039/C9AN01366K. 31799553

[ref27] Furikado I.; Forsman J.; Nylander T. Particle Adsorption Using a Quartz Crystal Microbalance with Dissipation by Applying a Kelvin-Voigt-Based Viscoelastic Model and the Gauss-Newton Method. Anal. Chem. 2023, 95, 15286–15292. 10.1021/acs.analchem.3c02642. 37782503

[ref28] Huang K.; Szlufarska I. Friction and slip at the solid/liquid interface in vibrational systems. Langmuir 2012, 28, 17302–17312. 10.1021/la303381z. 23157613

[ref29] Jana S.; Bhalla N. Acoustic Fingerprinting and Nanoslip Dynamics of Biofilms. Adv. Funct. Mater. 2024, 35, 2414687 10.1002/adfm.202414687.

