# Supplementary material for: Plasmonic Geometry-Induced Viscoelastic Biocomplex Formation with Optical Concealment, Liquid Slips, and Soundscapes in Bioassays
Source: Anal Chem. 2025 Mar 25;97(13):7020–7. doi: 10.1021/acs.analchem.4c04859 (PMC11983360; doi:10.1021/acs.analchem.4c04859)
Supplement: Supplementary file 2 — ac4c04859_si_002.pdf [file ac4c04859_si_002.pdf]

Supporting Information

Plasmonic geometry-induced viscoelastic biocomplex formation with optical concealment, liquid slips and soundscapes in bioassays

Zoe Bradley[a] and Nikhil Bhalla[a*]

[a]Nanotechnology and Integrated Bioengineering Centre (NIBEC), School of Engineering, Ulster University 2-24 York Street, Belfast, BT15 1AP, United Kingdom

* Corresponding author e-mail: n.bhalla@ulster.ac.uk

Table of Contents

Bioassay characterisation *S2*

QCM surface characterisation *S2*

QCM-D frequency shift with linear regression analysis *S4*

Charge measurements.. *S4*

Statistical analysis.. *S5*

Limit of Detection *S5*

Information on audio files.. *S6*

Bioassay characterisation

Figure S1a-b shows the characterisation of NPs in three different states: bare, carboxylated, and conjugated. The UV-Vis spectra highlight how the optical properties of NPs are influenced by surface modifications. Specifically, the carboxylation of AuNPs results in a minor red shift of 2 nm in their absorption peak, indicating a slight change in the local environment or electronic structure of the nanoparticles. AuNSts exhibit a more pronounced red shift of 88 nm upon carboxylation. This substantial shift suggests a significant change in the plasmon resonance of the AuNSts, due to their more complex geometry and increased surface area compared to AuNPs. For both NPs, the subsequent conjugation step indicates the addition of a protein peak at 280 nm. SEM images in Figure S1c-d indicate a monodispersed state for both NPs.

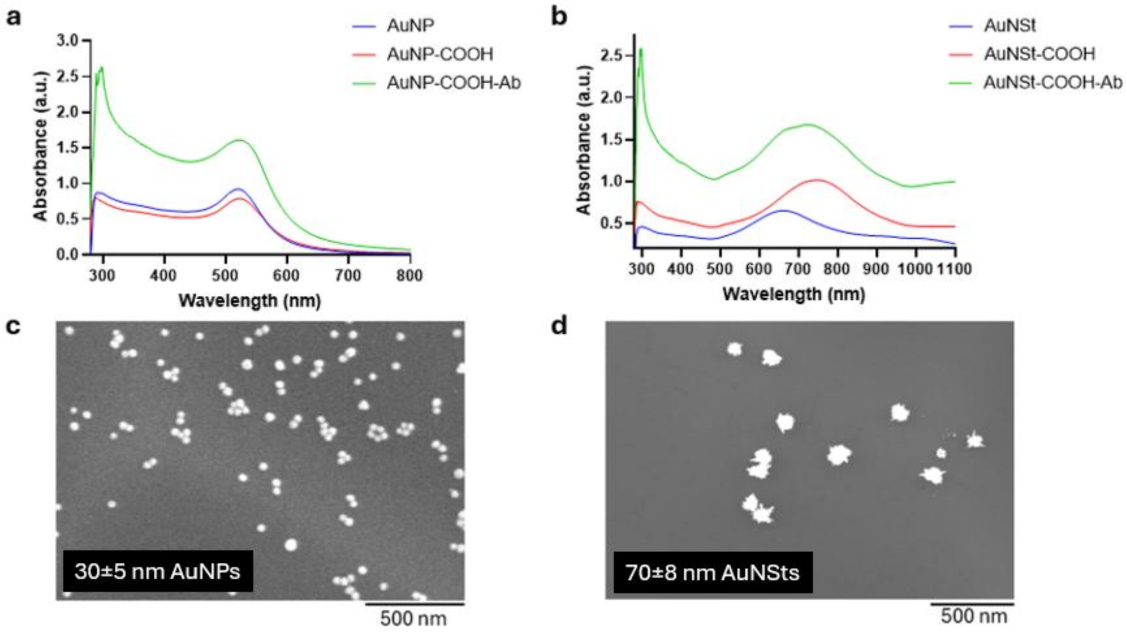

*Figure S1: UV-Vis and SEM characterisation **a** bare, carboxylated and conjugated AuNPs, **b** bare, carboxylated and conjugated AuNSts, **c** AuNPs and **d** AuNSts.*

QCM surface characterisation

AT-cut piezoelectric quartz crystals vibrating at 10 MHz are particularly well-suited for liquid biosensing due to their frequency stability and sensitivity to mass changes for accurate detection of biomolecular interactions. For quartz crystal characterisation, Figure S2a displays the SEM image of a bare quartz crystal surface, which reveals the presence, distribution and morphology of Au nano structures on the quartz crystal. Figure S2b presents an element composition spectrum from energy-dispersive X-ray spectroscopy, confirming that Au is the predominant element on the quartz surface. The surface has been polished with titanium, indicated by the presence of a small Ti peak.

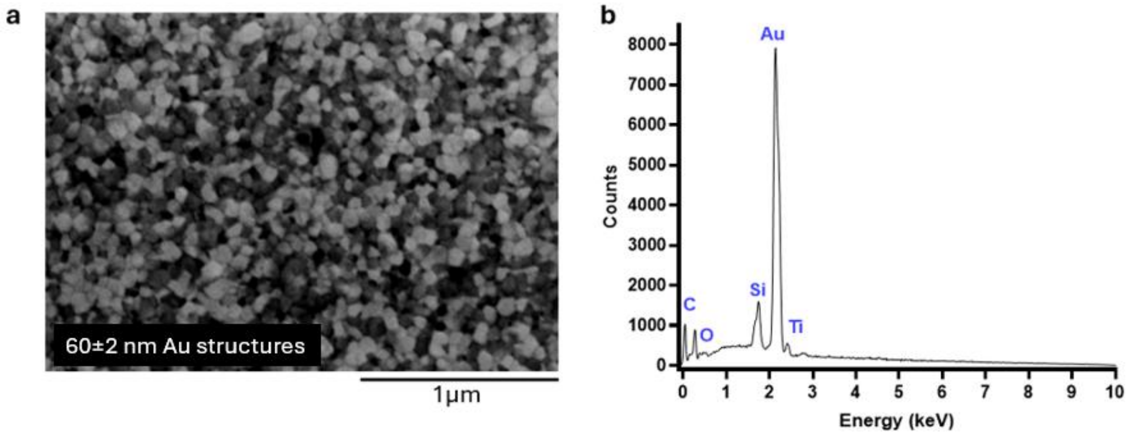

Figure S2: Bare quartz crystal surface characterisation. ***a*** *SEM image and* ***b*** *element composition spectrum.*

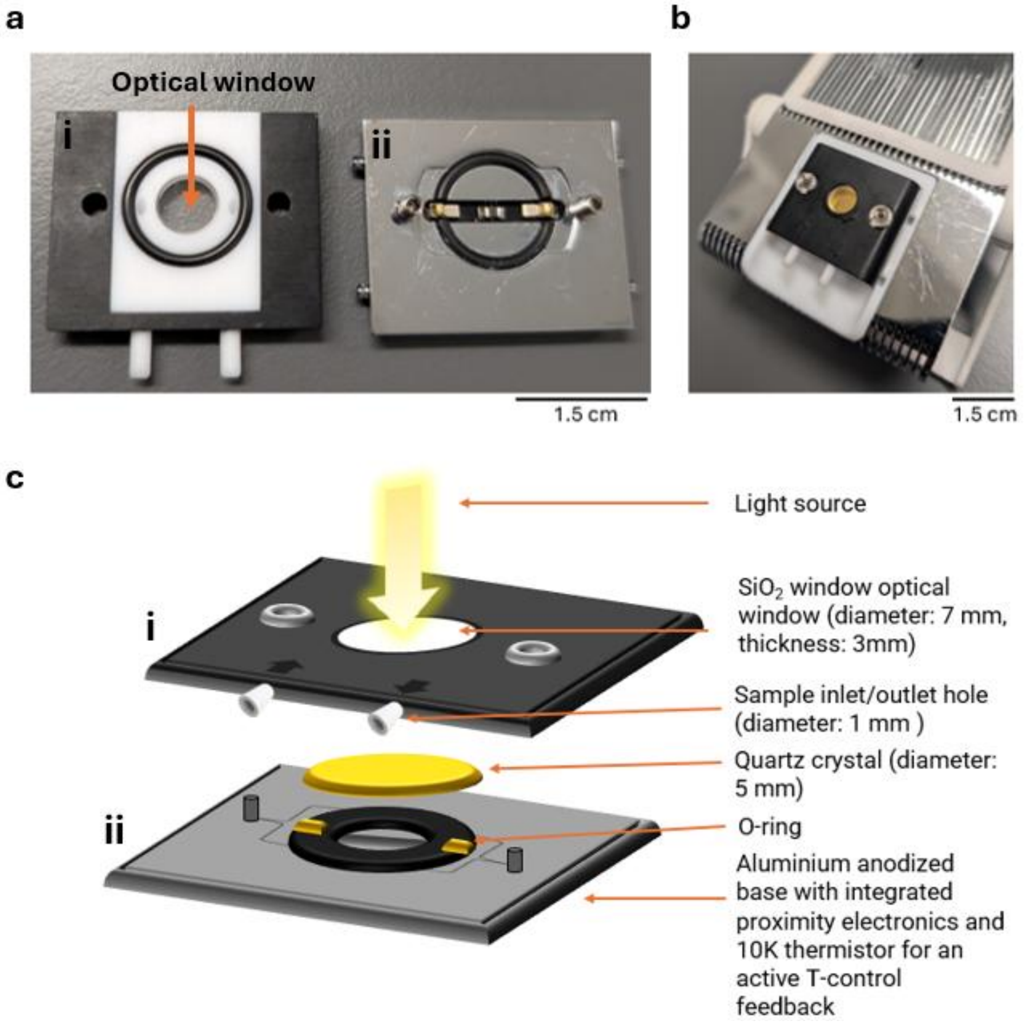

Figure S3: The quartz crystal casing, made of an anodized aluminium shell with a SiO_2 *optical window, enables the integration of LSPR.* ***a*** *Top and bottom parts of the casing,* ***b*** *closed casing with the quartz crystal sandwiched between the top and bottom parts of the casing, and* ***c*** *schematic showing the quartz crystal casing with the crystal and its dimensions. Part i represents the top of the casing, while part ii represents the bottom, as shown in* ***a*** *and* ***c****.*

As depicted in Figure S3c, the cylindrical inlet and outlet channels (1 mm in diameter) allow the sample to enter and exit the QCM-D module, interacting with the quartz crystal during measurements. The QCM crystal is sandwiched between parts i and ii, as shown in Figures S3a and S3c. The optical window in part i enables LSPR integration by allowing light to shine directly onto the quartz crystal. The anodized aluminium shell encompasses the bottom part of the QCM casing, as seen in part ii.

QCM-D frequency shift with linear regression analysis

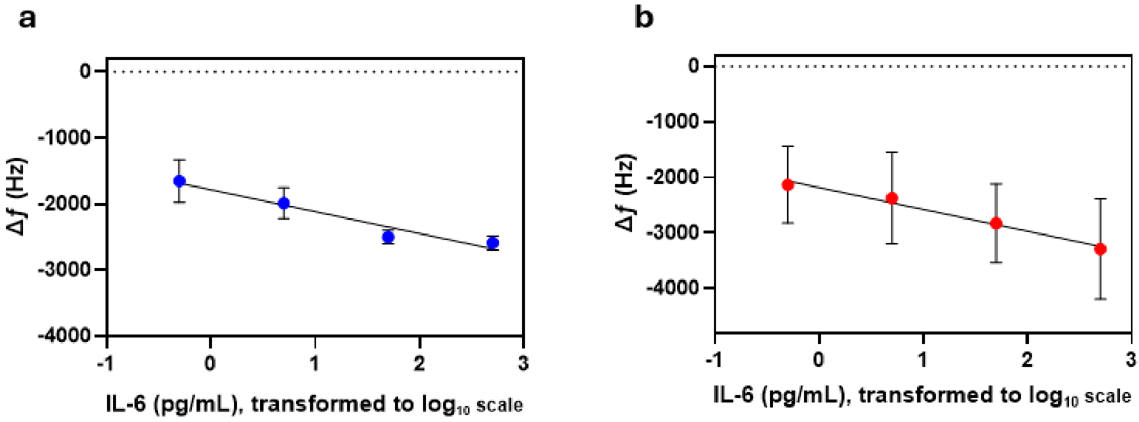

*Figure S4: QCM-D frequency and dissipation shifts displayed with linear regression analysis **a** AuNPs average frequency shifts on QCM-D surface and **b** AuNSts average frequency shifts on QCM-D surface.*

Charge measurements

Zeta potential measurements indicate the magnitude of the NP surface charge. A more negative the zeta potential, displayed by AuNPs indicates a greater electrostatic repulsion between particles, which leads to better NP stability and less aggregation, compared to AuNSts. Also, the more complex, star-shaped structure of AuNSts may result in less even surface coverage of functional groups on their surface, which can affect zeta potential.

Table S1: Zeta potential measurements obtained from DLS.

Sample	Zeta potential (mV)
10 mM PBS pH 7.4	-8.61 ±0.364
Capture Ab + Antigen	0.0994 ±0.136
AuNPs bioconjugate	-11.7 ±0.558
AuNSts bioconjugate	-10.3 ±0.207

Statistical analysis

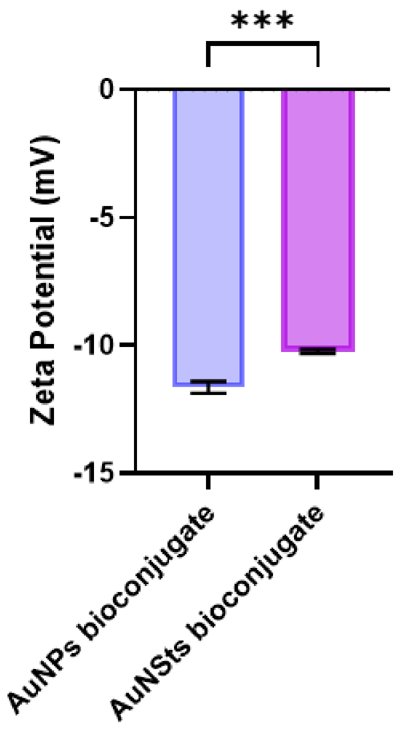

Figure S5: Comparisons of nanoparticle bioconjugates were conducted using a standard one-tailed t-test at a 95% confidence level. The significance level is indicated by the number of asterisks (*), with more asterisks representing greater significance. Error bars represent the standard deviation based on multiple experimental replicates used in the test, with n≥6.

Limit of Detection

The Limit of Detection (LOD) of the QCM-D experiment using AuNPs and AuNSts was calculated using the below equations:

Sensitivity is calculated from graph S4 where linear regression analysis uses the following equation:

$$y = mx + c$$

Where y is frequency shift (Hz), m is the sensitivity (Hz/pg/mL), x is IL-6 (pg/mL), transformed to $\log_{10}$ and C is the y-intercept. From this, the LOD is calculated using the below equation:

$$LOD = \frac{3\ x\ standard\ deviation\ of\ baseline\ frequency\ shift}{Sensitivity}$$

Information on audio files

Audio files extracted from the periodogram soundscapes for AuNPs (C1, C2, C3, C4) and AuNSts (C1, C2, C3, C4) correspond to IL-6 concentrations of 0.5, 5, 50, and 500 pg/mL, respectively.
